# Hydrothermal Synthesis and Nanoscale Characterization of Single-Unit-Cell Bi_2_WO_6_ Nanosheets: Correlation between Morphology, Adhesion, and Surface Potential

**DOI:** 10.1021/acsomega.6c05132

**Published:** 2026-07-07

**Authors:** Charles Duarte Almeida, Beatriz da Silva Batista, Madson Emanuel Vieira Mendonça, Dyego Maia de Oliveira, Eduardo Padrón Hernández, Clenilton Costa dos Santos, Alan Silva de Menezes, Luciana Magalhães Rebelo Alencar, João Victor Barbosa Moura

**Affiliations:** † Programa de Pos-graduacao em Fisica, 37892 Universidade Federal do Maranhao, Sao Luis 65080-805, MA, Brasil; ‡ Laboratorio de Biofísica e Nanosistemas, Universidade Federal do Maranhao, Sao Luis 65080-805, MA, Brasil; § Departamento de Fisica, 28116 Universidade Federal de Pernambuco, Recife 50740-540, PE, Brasil

## Abstract

Single-unit-cell (SUC) Bi_2_WO_6_ nanosheets were successfully synthesized via a hydrothermal route and investigated through a comprehensive nanoscale characterization. Structural analyses by powder X-ray diffraction, Raman spectroscopy, and transmission electron microscopy confirmed the formation of a polycrystalline two-dimensional material. Atomic force microscopy revealed uniform nanosheets with an average thickness of ∼1.42 nm, consistent with a single-unit-cell configuration. Nanoscale mechanical analysis demonstrated that increasing PeakForce (1–5 nN) induces irreversible deformation of pre-existing surface pores, while higher oscillation frequencies (2–4 kHz) promote additional topographic modifications, indicating a frequency-dependent surface stability. Kelvin probe force microscopy (KPFM) mapping revealed a pronounced spatial variation in surface potential, with a lower work function at the nanosheet center and higher values at the edges. This edge-center electronic contrast establishes a direct correlation between morphology, adhesion behavior, and local surface potential, providing new insights into charge distribution in SUC-Bi_2_WO_6_. These findings highlight the relevance of nanoscale heterogeneity in governing surface reactivity, reinforcing the potential of Bi_2_WO_6_ nanosheets for applications in photocatalysis and nanoelectronics.

## Graphical abstract

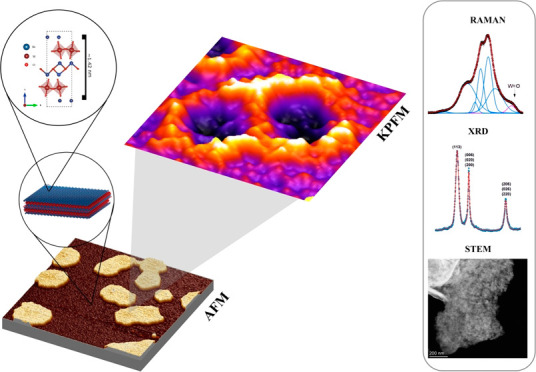

## 1 Introduction

Two-dimensional (2D) materials exhibit distinct physical and chemical properties compared to their bulk counterparts.^1–3^ Since the discovery of graphene, interest in 2D materials has grown exponentially and expanded to include layered oxides, such as bismuth tungstate (Bi_2_WO_6_), due to their potential in photocatalysis, energy storage, electrocatalysis, and organic catalysis.^4–6^

Bi_2_WO_6_ is an n-type oxide semiconductor from the Aurivillius family. These materials follow the general formula: Bi_2_A_n‑1_B_n_O_3n+3_ or equivalently (Bi_2_O_2_)­(A_n‑1_B_n_O_3n+1_), where A can be a metal such as Na, K, Ca, Sr, Ba, Pd, and Bi and B is a transition metal such as Ti, Nb, Ta, Mo, W, and Fe, typically forming layered structures.^7^ Bi_2_WO_6_ adopts an orthorhombic crystal structure consisting of alternating (Bi_2_O_2_)_n_^2n+^ and perovskite-like (WO_4_)_n_^2n–^ layers, where oxygen atoms bridge these layers through chemical bonds.^8^

This layered arrangement generates an internal electric field that promotes spatial charge separation, making Bi_2_WO_6_ a promising photocatalyst active in visible light. In its single-unit cell, bismuth tungstate (SUC-Bi_2_WO_6_) nanosheets are particularly notable for their efficient separation of electron–hole pairs, short charge transport paths, high specific surface area, and high adsorption capacity.^7,9–11^ A deep understanding of Bi_2_WO_6_ at the nanoscale is crucial for optimizing its performance in photocatalysis, electronic devices, and optoelectronics, where charge transfer and surface potential are key factors.

Comprehensive analysis of 2D materials requires advanced techniques, such as atomic force microscopy (AFM) and Kelvin probe force microscopy (KPFM), which enable investigation of their nanoscale surface interactions and surface potential.^12^ Such techniques have been extensively employed to characterize 2D materials like tungsten diselenide (WSe_2_), molybdenum disulfide (MoS_2_), and molybdenum diselenide (MoSe_2_).^1,13,14^ Despite significant advances in the synthesis of SUC-Bi_2_WO_6_ nanosheets, most studies focus on the average charge transfer in Bi_2_WO_6_ heterojunctions, while the local surface potential of isolated single-unit-cell nanosheets remains unexplored.

This study investigates the structural, morphological, and electronic properties of SUC-Bi_2_WO_6_ nanosheets synthesized hydrothermally, using powder X-ray diffraction (PXRD), transmission electron microscopy (TEM), raman spectroscopy (RS), AFM, and Kelvin probe force microscopy (KPFM). PXRD, TEM, and Raman spectroscopy provide insights into the material’s crystallographic structure and vibrational modes. AFM provides detailed information on its morphology and surface interactions, specifically focusing on adhesion forces under varying scanning conditions. KPFM enables high-resolution mapping of surface potential, revealing the charge distribution on the surface of SUC-Bi_2_WO_6_ nanosheets. The integrated approach provides a comprehensive insight into the properties of SUC-Bi_2_WO_6_ in its two-dimensional configuration. This systematic nanoscale characterization provides a robust framework for investigating 2D materials, establishing a foundation for future research in the field.

## 2 Materials and Methods

### 2.1 Synthesis

The preparation of layered Bi_2_WO_6_ nanosheets was achieved through hydrothermal synthesis, following the methodology outlined in a previous publication.^15^ Sodium tungstate dihydrate [Na_2_WO_4_·2H_2_O] (≥99%, Sigma-Aldrich, St. Louis, MO, USA), bismuth nitrate pentahydrate [Bi­(NO_3_)_3_·5H_2_O] (≥98.0%, Sigma-Aldrich, St. Louis, MO, USA), and hexadecyltrimethylammonium—CTAB [CH_3_(CH_2_)_15_N­(Br)­(CH_3_)_3_] were employed as initial precursors. For the synthesis, 1 mmol of Na_2_WO_4_·2H_2_O, 2 mmol of Bi­(NO_3_)_3_·5H_2_O, and 0.05 g of CTAB were dissolved in 80 mL of deionized water. The aqueous solution was stirred for 30 min at an average speed of 1500 rpm, then transferred to a 100 mL Teflon-lined stainless autoclave, and maintained at 120 °C for 24 h. The resulting white precipitates were washed repeatedly with deionized water and dried in an oven at 60 °C for 10 h.

### 2.2 Sample Characterization

#### 2.2.1 Powder X-ray Diffraction

The layered Bi_2_WO_6_ nanosheets were structurally characterized by Powder X-ray diffraction, using the Bruker D8 Discover diffractometer, equipped with the linear LynxEye XE detector and CuKα radiation (λ = 1.5418 Å) operating at 40 kV/40 mA. The measurement was performed in the 2θ range of 20°–100°, with a step size of 0.02° and a counting time of 2 s/step. The structural parameters of the Bi_2_WO_6_ sample were obtained by the Rietveld refinement^16^ using the crystal data in the Inorganic Crystal Structure Database (ICSD)—Card. No. 66579.^17^ The Rietveld refinement was performed using the GSAS II software.^18^

#### 2.2.2 Raman Spectroscopy

Raman spectra were acquired using a Horiba T64000 spectrometer that features a liquid-nitrogen-cooled CCD detector. The excitation source was a 532 nm laser. A microscope (Olympus BX41) equipped with a 20.5 mm focal length objective lens and a numerical aperture of 0.35 was used to focus the laser on the sample surface. The spectrometer slits were adjusted to achieve a resolution of 2 cm^–1^. Each spectrum was obtained by averaging four accumulations, with an integration time of 60 s per accumulation. The laser power was maintained at a low level to prevent the local heating of the sample.

#### 2.2.3 Transmission Electron Microscopy

TEM was performed on a TALOS 200Fi-Thermo Fisher microscope operating at 200 kV, equipped with a CETA 4k camera, and a HAADF detector. Morphological and structural analyses were carried out by HRTEM, STEM, and HRSTEM.

#### 2.2.4 Atomic Force Microscopy

Bismuth tungstate samples were characterized by AFM using a Multimode 8 instrument (Bruker, USA) equipped with a controller (NanoScope V). For this purpose, the nanosheets were dispersed in acetone via ultrasonication and subsequently deposited onto freshly cleaved mica substrates. The characterization was carried out using two AFM modes: Peak Force Quantitative Nanomechanical Mapping (QNM) and Kelvin Probe Force Microscopy (KPFM), which provided simultaneous topographic, adhesion, and surface potential data. For QNM measurements, a ScanAsyst-Air probe (Bruker) with a nominal tip radius of 4 nm and a nominal spring constant of 0.4 N/m was used. For KPFM measurements, a PFQNE-AL probe (Bruker) with a nominal tip radius of 5 nm and a nominal spring constant of 0.8 N/m was employed. KPFM measurements were performed in Frequency Modulation (FM) mode using the lift technique under a sample bias configuration. The lift height was selected based on experimental trials and set to 35 nm to ensure the elimination of topographic interference while maintaining a high signal-to-noise ratio and preserving the lateral resolution at the nanosheet edges. The actual spring constants of both probes were calibrated using the thermal tune method.^19,20^ Additionally, to enable work function calculations, the KPFM tip (Φt_ip_) was calibrated prior to the measurements using a standard Al–Si–Au grid, yielding a value of 4.601 eV. A detailed description of this calibration procedure is provided in the Supporting Information

A systematic parameter study was conducted, investigating the effect of the PeakForce tapping frequency (1, 2, and 4 kHz) and PeakForce (1, 2, 3, 4, and 5 nN) on the properties obtained. All experiments were performed under ambient conditions (23 °C and 44% relative humidity). All AFM maps were acquired with a high spatial resolution of 256 × 256 pixels. Topography, adhesion, and surface potential were extracted and analyzed according to established methodologies reported in the literature.^19–21^ Data postprocessing and analysis were performed using Gwyddion (v. 2.63)^22^ and Nanoscope Analysis (v. 2.0) software.

## 3 Results

### 3.1 Powder X-ray Diffraction

To determine the structure of the SUC-Bi_2_WO_6_ layer, the samples were analyzed by powder X-ray diffraction (PXRD). The structural parameters were determined using the Rietveld refinement. Figure 1 presents the X-ray diffractogram with Rietveld refinement, demonstrating a strong correlation between the observed and calculated PXRD patterns. The results confirm a well-crystallized orthorhombic structure in the P2_1_ab space group, with lattice parameters a = 5.464(5) Å, b = 5.470(5) Å, c = 16.364(4) Å, and unit cell volume of 489.2(1) Å^3^. These values show excellent agreement with the parameters of ICSD no. 66579 ^17^. No evidence of a secondary phase was detected, indicating the high purity of the synthesized material.

**Figure 1:**
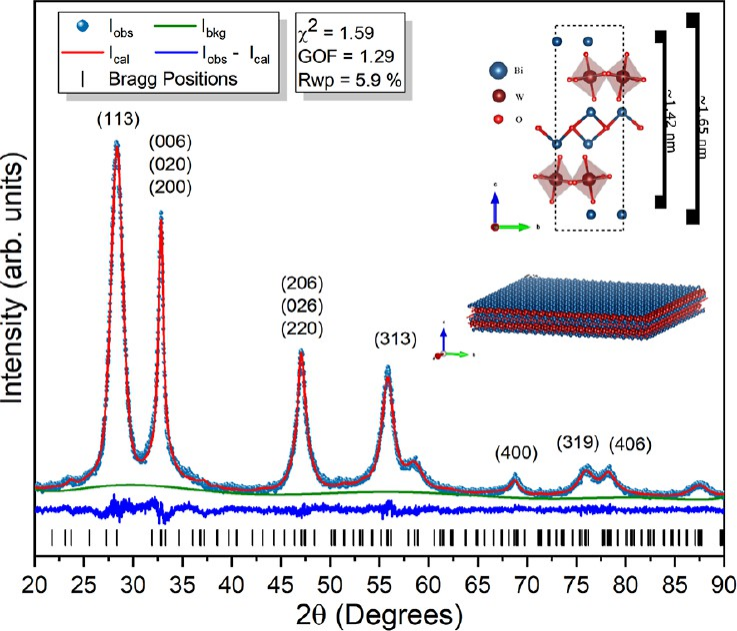
Powder X-ray diffraction pattern of the SUC-Bi_2_WO_6_ layer obtained by hydrothermal synthesis. Insert: Representation of the crystal structure of the orthorhombic phase Bi_2_WO_6_ at room temperature viewed from the a-axis, showing the distorted and edge-shared WO_6_ octahedra (W—Brown, Bi—Dark Blue, and O—Red atoms). (For interpretation of the references to color in this figure legend, the reader is referred to the Web version of this article.)

The diffractogram exhibits broad peaks showing that the sample has nanometric crystallite size. The (113) reflection exhibits a broader peak than the (200)/(020)/(006) reflections, evidencing an anisotropy in the crystallite size or microstrain. In layered materials such as Bi_2_WO_6_, this effect is typically more pronounced along the c-axis, resulting in crystallites that are smaller in this direction compared to the a and b axes. The best Rietveld refinement results were obtained considering a crystallite size anisotropy, using the [001] direction as the anisotropy axis, and an isotropic microstrain. The obtained crystallite sizes parallel D_||_ and perpendicular D_⊥_ to the [001] axis are 27.3(1) nm and 3.7(1) nm, respectively. The obtained microstrain value is 1.94(2) %. These results are consistent with the plate-like morphology observed by TEM and AFM, as discussed in a subsequent section.

### 3.2 Raman Spectroscopy

The room-temperature Raman spectrum of the single-unit-cell Bi_2_WO_6_ layer is displayed in Figure 2. This highly sensitive technique was employed to investigate the structure and identify the characteristic vibrational modes. Extending from 70 to 1000 cm^–1^, the spectra exhibit broad and partially overlapping Raman peaks, giving rise to shoulder features that hinder clear peak distinction.

**Figure 2:**
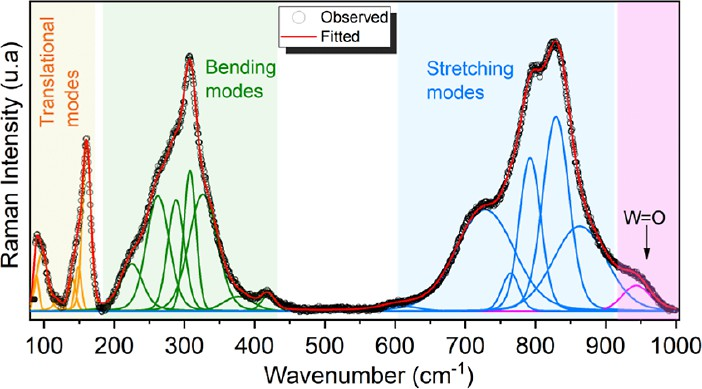
Raman spectrum of a SUC-Bi_2_WO_6_ nanosheet acquired at room temperature. The experimental data (black circles) were fitted using Pseudo-Voigt functions (red line). Colored peaks indicate distinct vibrational modes characteristic of the structure. The magenta shaded region highlights the W = O stretching mode, which serves as a spectroscopic marker of the 2D Bi_2_WO_6_ structure.

The peaks observed below 200 cm^–1^ were attributed to the translational mode involving simultaneous T́(Bi) and T́(W) units motions. The Raman modes observed at 224, 262, 288, and 307 cm^–1^ are assigned to the bending vibrations of WO_6_ octahedra involving apical oxygen atoms (O1 and O6). The bands at 326 and 376 cm^–1^ are related to the bending vibrations of the (Bi_2_O_2_)^2+^ layers, while the modes at 418 and 605 cm^–1^ correspond to the bending vibrations of WO_6_ units involving equatorial oxygen atoms (O4 and O5). The Raman peaks at 728, 764, and 792 cm^–1^ are attributed to the antisymmetric stretching vibrations of WO_6_ (equatorial oxygens), whereas the band at 829 cm^–1^ is assigned to the symmetric stretching of WO_6_ (apical oxygens).^23–25^ Finally, the mode at 863 cm^–1^ corresponds to an antisymmetric stretching of WO_6_ involving apical oxygens,^25^ and the high-frequency band at 953 cm^–1^ is associated with a terminal W = O stretching vibration, which appears exclusively in the 2D Bi_2_WO_6_ structure.^26^ These results, particularly the presence of the W = O stretching mode above 953 cm^–1^, serve as a marker of the two-dimensional structure and confirm the successful formation of the bismuth tungstate sample in the form of a single-unit-cell layer.

### 3.3 Transmission Electron Microscopy

The morphology and atomic structure of the SUC-Bi_2_WO_6_ layers were analyzed using TEM. The uniform structure of the SUC-Bi_2_WO_6_ layer was confirmed by STEM images, which revealed the formation of ultrathin nanosheets with a lamellar morphology (Figure 3a). The selected-area electron diffraction (SAED) pattern (Figure 3b) shows well-defined concentric rings indexed to the (113) and (200)/(020) planes, confirming the polycrystalline nature of SUC-Bi_2_WO_6_. High-resolution TEM (HRTEM) images (Figure 3c) showed an interplanar distance of 2.71 Å across the lamella surface, consistent with structural characterizations. This spacing corresponds to half of the lattice parameters a (5.457 Å) or b (5.436 Å), where this observation is consistent with the 2D morphology of the crystallites, which are extended along the a–b plane and exhibit a reduced dimension along the c-axis. The ultrathin nanosheets shown in Figure 3a are formed by several crystallite domains, like the one shown in Figure 3c, and their size is consistent with the size obtained by PXRD and Rietveld refinement. High-resolution STEM (HRSTEM) images (Figure 3d) show lattice fringes with a spacing of 3.15 Å, corresponding to the (113) plane.^27^ This confirms the high crystallinity and structural order of the SUC-Bi_2_WO_6_ layers, in agreement with the PXRD and Raman results.

**Figure 3:**
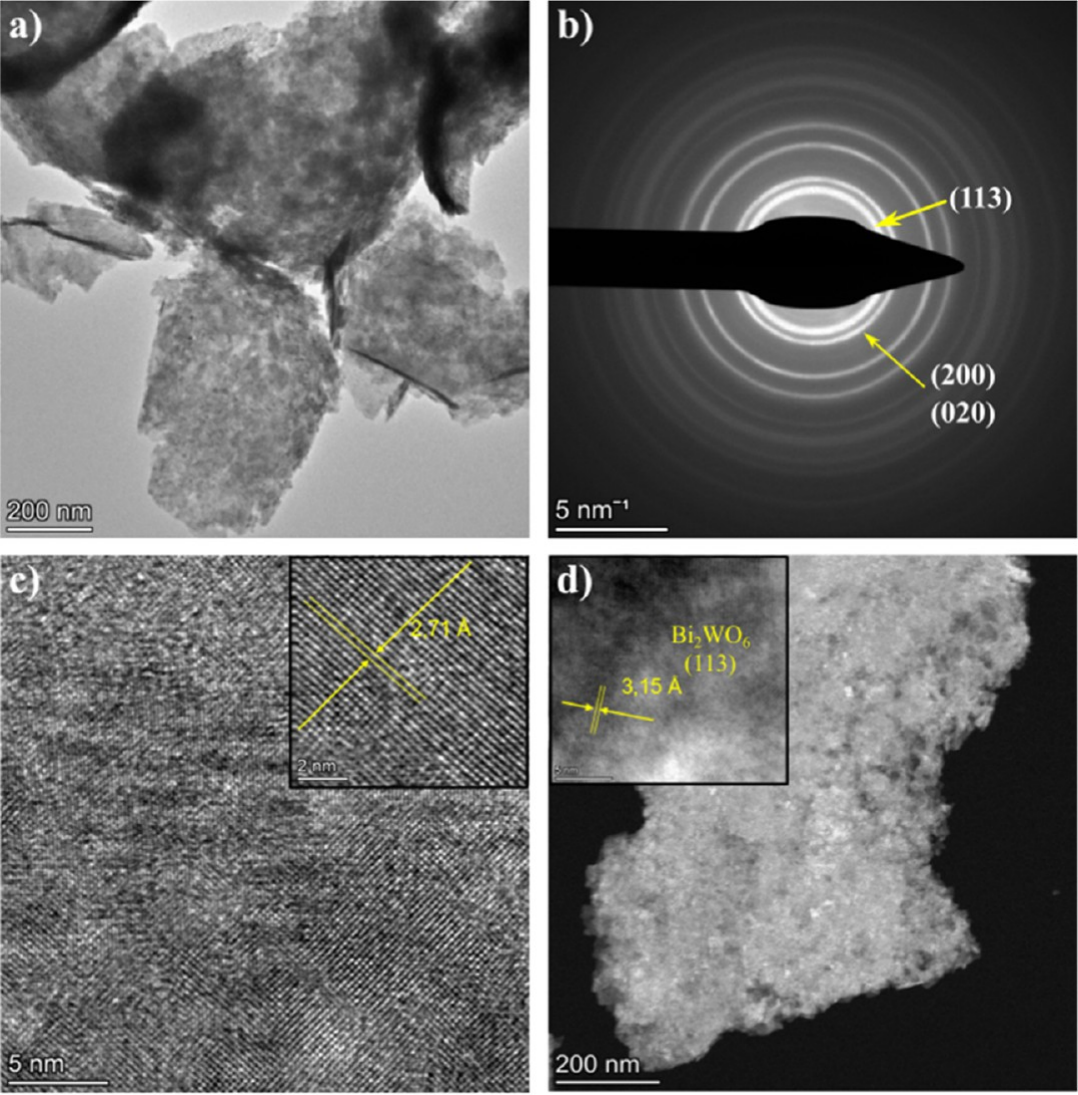
TEM and HRTEM of the single-unit-cell Bi_2_WO_6_ layer. (a) TEM image showing nanosheets with a lamellar morphology. (b) SAED pattern showing concentric diffraction rings, indicative of a polycrystalline structure. (c) HRTEM image revealing lattice fringes with an interplanar spacing of 2.71 Å, corresponding to the (200)/(020) planes. (d) HRSTEM image, showing high-resolution lattice fringes with 3.15 Å spacing assigned to the (113) plane.

### 3.4 Characterization via AFM

AFM topography maps revealed well-defined Bi_2_WO_6_ nanosheets deposited on freshly cleaved mica substrates. Figure 4a,b shows the two-dimensional (2D) and three-dimensional (3D) scan of a 2.5 × 2.5 μm^2^ area. In these maps, brighter regions correspond to SUC-Bi_2_WO_6_ layers, while darker areas indicate the exposed mica substrate. These maps reveal discrete nanosheets with uniform heights, suggesting the presence of individual SUC layers separated by well-defined boundaries.

**Figure 4:**
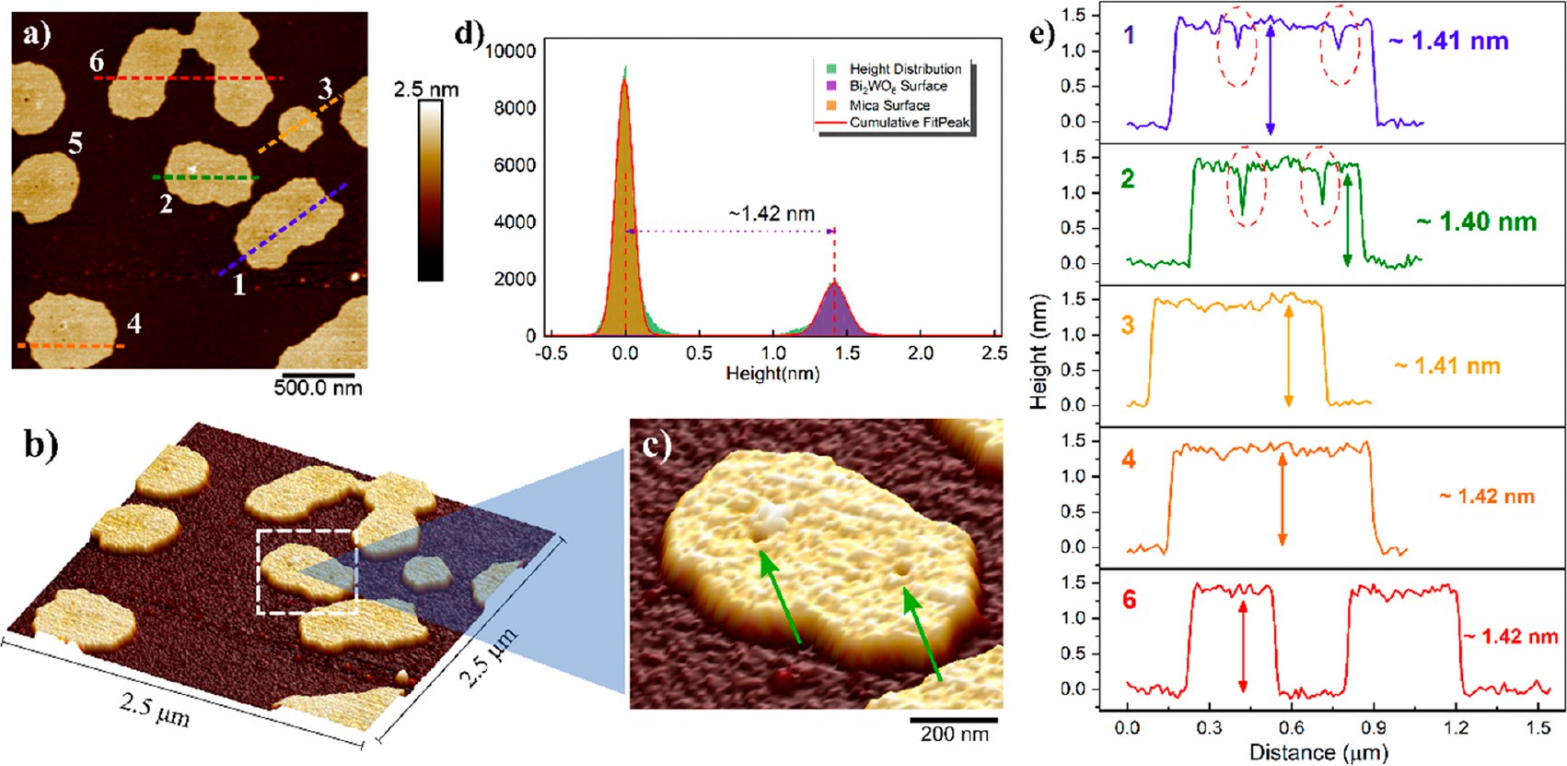
AFM measurements of the SUC-Bi_2_WO_6_ layer. (a) A 2D topographic map with 2.5 μm × 2.5 μm and (b) a 3D view. The white dotted square demarcates the magnification region shown in the image (c), highlighting the surface of a SUC-Bi_2_WO_6_ layer. The green arrows indicate the holes present on the surface. (d) Histogram distribution of height values of the topographic map. Cross-sectional profiles in (e) correspond to the dashed lines in Figure (a), illustrating the thickness of different nanosheets with red dashed circles marking holes detected along the profile lines.

The AFM analysis revealed that the SUC-Bi_2_WO_6_ nanosheets exhibit morphologies varying from nearly circular to slightly elongated. The height histogram (Figure 4d) derived from topographic maps displays two distinct peaks: one at −0.007 ± 0.001 nm, corresponding to the mica surface, and another at 1.410 ± 0.002 nm, associated with the SUC-Bi_2_WO_6_ nanosheets, yielding an average thickness of 1.420 ± 0.002 nm. Cross-sectional profiles (Figure 4e) taken along selected nanosheets, indicated by the dashed lines, exhibit lateral dimensions of approximately 600 nm and reveal a homogeneous thickness with an average value of 1.42 nm, which aligns closely with the expected value for a single-unit-cell layer. Localized depressions along the profiles, indicated by red dashed circles in Figure 4e, are associated with surface holes. These features are better visualized in the magnified topographic image of an individual nanosheet (Figure 4c), where holes are marked by green arrows.

This thickness measured by AFM (≈1.42 nm) exhibits a strong correlation with the out-of-plane lattice parameter c = 1.64 nm obtained by PXRD, which corresponds to the interlayer spacing along the [001] direction of the orthorhombic Bi_2_WO_6_ structure. The slight difference arises from the nature of each technique: AFM measures the physical height of individual nanosheets, which may be affected by surface adsorbates, substrate interactions, or partial dehydration under ambient conditions, while PXRD provides an average interplanar spacing that includes van der Waals gaps and reflects structural periodicity. Additionally, few-layer systems may undergo out-of-plane relaxation, leading to slightly reduced thicknesses. The agreement of these complementary measurements confirms that the observed nanosheets are indeed single-unit-cell Bi_2_WO_6_ layers, highlighting the importance of combining morphological and crystallographic techniques to understand the structure of two-dimensional materials better.

The direct formation of single-unit-cell Bi_2_WO_6_ nanosheets represents an important distinction from many ultrathin-material preparation strategies reported in the literature. In layered van der Waals materials, ultrathin sheets are commonly obtained through exfoliation processes that typically generate a distribution of layer thicknesses, including multilayer and few-layer structures.^28–30^ In contrast, Bi_2_WO_6_ does not possess weak interlayer van der Waals bonding that would allow conventional exfoliation. The present hydrothermal synthesis directly produces nanosheets with thicknesses corresponding to a single crystallographic unit cell, as confirmed by AFM, PXRD, Raman spectroscopy, and TEM analyses. This direct growth mechanism enables the production of a homogeneous powder composed predominantly of single-unit-cell nanosheets without requiring additional delamination or thickness-selection procedures, which is advantageous for both fundamental studies and future applications involving two-dimensional oxide materials.

In the literature, different thicknesses have been reported for Bi_2_WO_6_ layers. Leandro et al.^31^ and Zhou et al.^15^ describe monolayers with a height of ∼ 0.8 nm, corresponding to half of the unit cell along the [001] direction. In contrast, other works report values around ∼ 1.6 nm, which match the thickness of a full unit cell, commonly referred to as “single-unit-cell layer”.^24,32^ The thickness observed in this work (∼1.4 nm) is in good agreement with these latter reports, indicating that the obtained nanosheets correspond to a single-unit cell.

In addition to height correspondence, detailed surface roughness analysis indicates a smooth topography, with an arithmetic mean roughness (S_a_) of 81 ± 6 pm and a root-mean-square roughness (S_q_) of 140 ± 11 pm. Although these parameters provide an overall measure of surface irregularity, they are not sensitive to the differentiation between peaks and valleys. Therefore, additional statistical descriptors were employed, such as asymmetry (S_sk_) and kurtosis (S_ku_), as summarized in Table 1 for the six analyzed SUC-Bi_2_WO_6_ layers.

**Table 1:** Statistical Parameters of Surface Roughness Maps Obtained from 2.5 μm × 2.5 μm Scans.

SUC-Bi_2_WO_6_	S_a_ (pm)	S_q_ (pm)	S_sk_	S_ku_	Area (μm^2^)
1	81.0	133.1	–3.13	17.77	0.306
2	84.0	149.9	–2.75	16.14	0.221
3	89.4	150.9	–3.05	12.6	0.072
4	73.2	127.6	–3.54	20.35	0.276
5	85.9	148.5	–3.74	22.36	0.184
6	74.5	129.7	–3.83	23.32	0.523
Mean ± SD	81 ± 6	140 ± 11	–3.3 ± 0.4	19 ± 4	0.3 ± 0.2

S_a_: average roughness, S_q_: quadratic average roughness, S_sk_: asymmetry, and S_ku_: kurtosis

The height distribution of the SUC-Bi_2_WO_6_ layer exhibited negative skewness (S_sk_ = −3.3 ± 0.4), indicating that the surface contains deeper valleys relative to the midline,^33,34^ consistent with the presence of nanoscale holes (highlighted in Figure 4b). The kurtosis value (S_ku_ = 19 ± 4) reveals a sharply peaked distribution, with most height values clustered around the mean and a small number of extreme values.^33,34^ This suggests a high degree of vertical homogeneity punctuated by localized features.

These findings confirm the formation of predominantly single-unit-cell Bi_2_WO_6_ domains, characterized by well-defined geometries and a uniform, topographically homogeneous surface. Building upon this morphological characterization, subsequent AFM measurements were performed to investigate the nanoscale surface and adhesive properties of SUC-Bi_2_WO_6_ nanosheets.

#### 3.4.1 Probe Oscillation Frequency Variation

A single nanosheet was selected for the investigation to explore the effect of the probe’s oscillation frequency on the behavior of SUC-Bi_2_WO_6_ nanosheets on mica. Scans were performed at frequencies 1, 2, and 4 kHz, enabling a comprehensive analysis of the interactions between the probe and the SUC-Bi_2_WO_6_ layer. This investigation provided data on morphological stability and adhesion forces behavior as a function of oscillation frequency.

Figure 5 shows the topography (a–c) and adhesion (d–f) maps for the single nanosheet acquired at different oscillation frequencies. In the adhesion maps, the color coding is used to distinguish areas of high adhesion (light orange tones) and low adhesion (dark purple tones). Compared to the SUC-Bi_2_WO_6_ layer, the mica substrate exhibits stronger interactions with the AFM probe, which is visually reflected by brighter tones. This color contrast enables a clear distinction between the substrate and the nanosheet. The adhesion signal is interpreted here in a relative sense, enabling comparison between the nanosheet and substrate regions.

**Figure 5:**
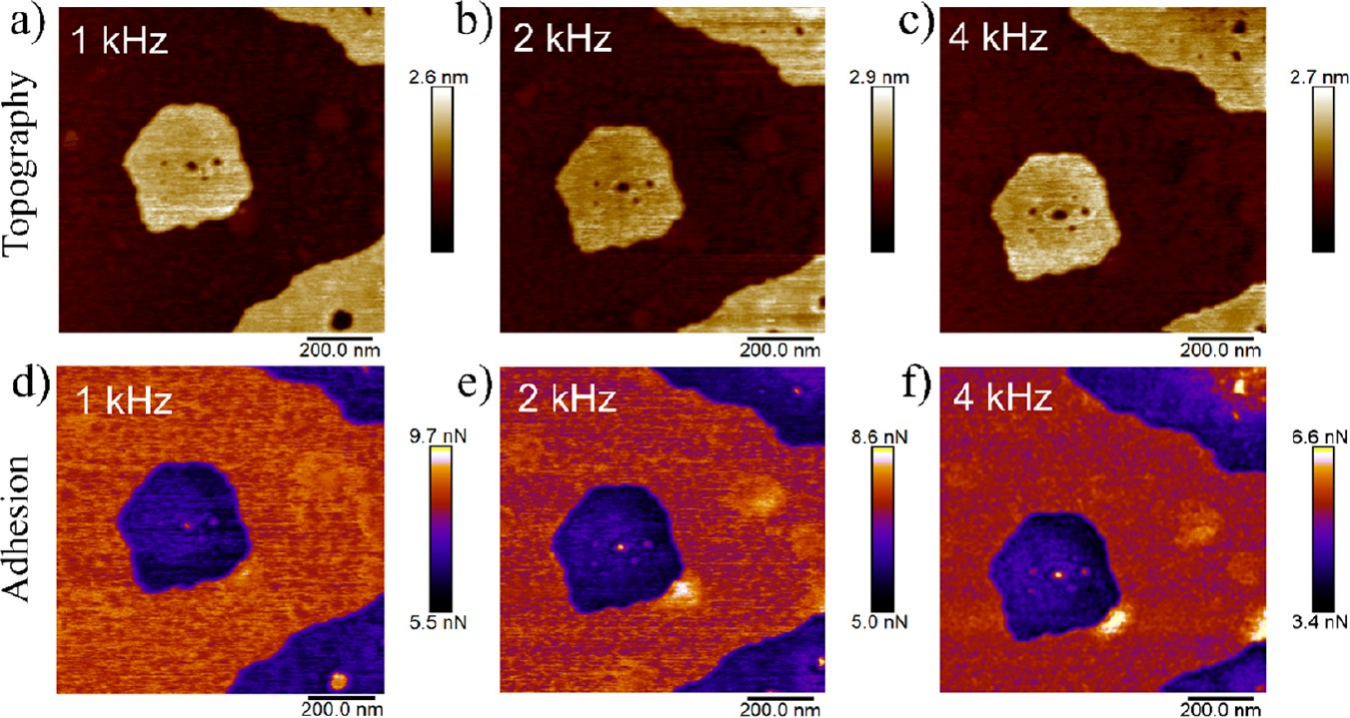
Two-dimensional maps of topography (a–c) and adhesion forces (d–f) of a SUC-Bi_2_WO_6_ layer nanosheet on mica, varying the oscillation frequency from 1 kHz to 4 kHz, with a square scanning area of 2.5 × 2.5 μm^2^.

The topographical maps (Figure 5a–c) reveal small holes on the nanosheet’s surface, whose number and diameter grow with increasing oscillation frequency. This progression leads the probe to interact with the underlying substrate. The adhesion maps (Figure 5d–f) support this interpretation, showing light-colored regions emerging at the center of the nanosheet as the frequency rises, suggesting increased exposure of the substrate below.

Figure 6 illustrates the statistical distribution of the adhesion values at different probe frequencies. The histogram analysis distinguishes the contributions of the SUC-Bi_2_WO_6_ nanosheets and the mica substrate. Notably, for a given surface (mica or SUC-Bi_2_WO_6_), the histograms present multiple Gaussian components. These components correspond to different interaction domains of the surface. However, as the oscillation frequency increases, a clear reduction is observed in both the average and dispersion. The distribution is narrow and shifts toward lower adhesion values, which consequently reduces the number of distinguishable Gaussian components due to a decreased contrast between these interaction domains.

**Figure 6:**
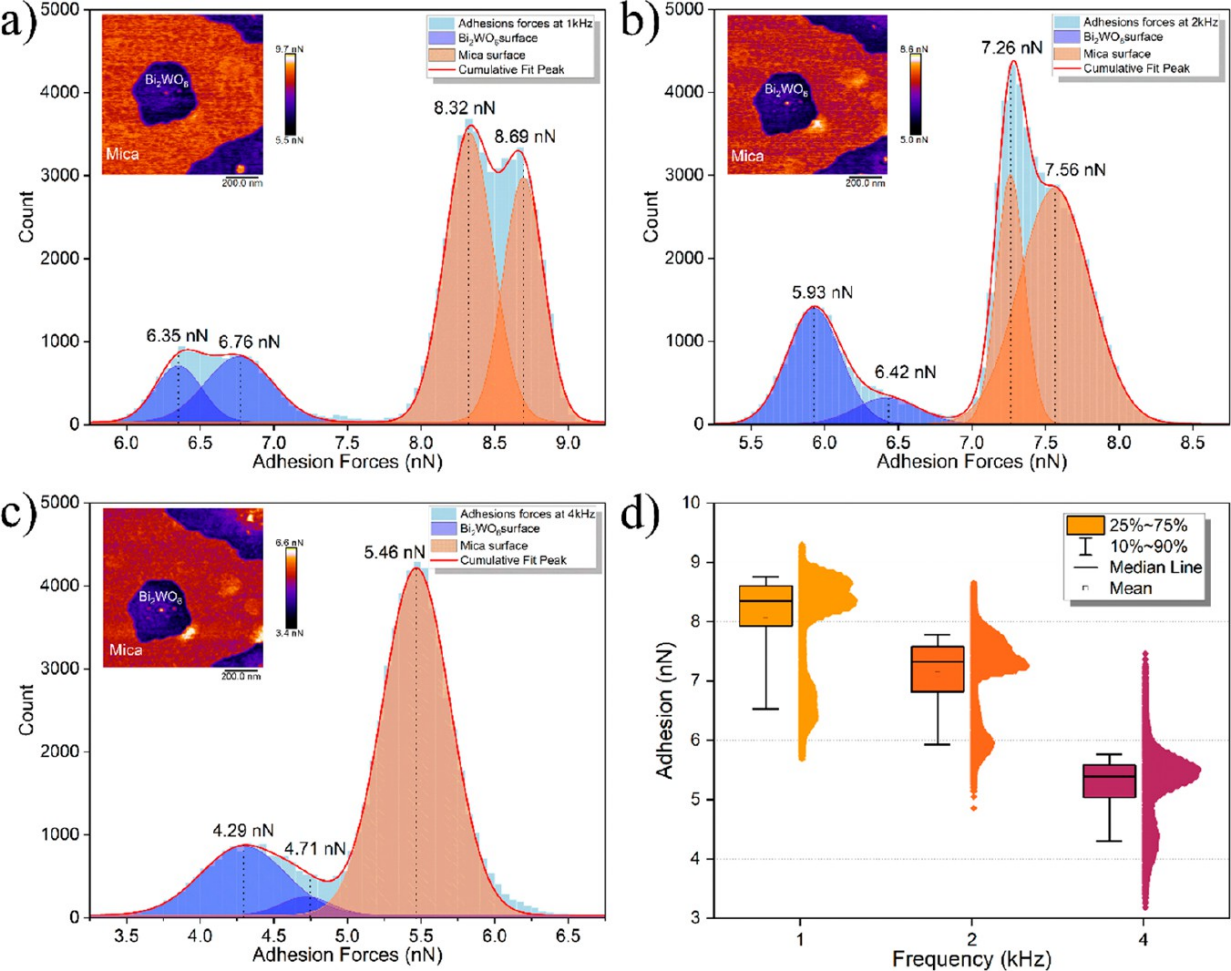
Adhesion forces histograms of the SUC-Bi_2_WO_6_ nanosheet on mica at different oscillation frequencies: (a) 1 kHz, (b) 2 kHz, and (c) 4 kHz, respectively, fitted with multiple Gaussian components, where the peaks correspond to distinct interaction domains. The blue components are associated with the SUC-Bi_2_WO_6_ nanosheet, while the orange components correspond to the mica substrate. Insets show the corresponding adhesion maps. (d) Statistical distribution of adhesion forces values as a function of frequency, indicating a systematic reduction in adhesion with increasing frequency.

For the mica substrate, adhesion values decrease from 8.330 ± 0.004 nN and 8.690 ± 0.004 nN at 1 kHz to 7.260 ± 0.001 nN and 7.560 ± 0.006 nN at 2 kHz and further reduce to 5.470 ± 0.001 nN, accompanied by a narrowing of the distribution. The SUC-Bi_2_WO_6_ surface displays a similar trend. Initial values of 6.35 ± 0.02 nN and 6.76 ± 0.03 nN at 1 kHz decrease to 5.930 ± 0.008 nN and 6.43 ± 0.04 nN at 2 kHz and further to 4.29 ± 0.04 nN and 4.71 ± 0.05 nN at 4 kHz. This indicates a reduction in the effective adhesion due to decreased interaction time between the probe and the sample.

To further evaluate the adhesion contrast, a relative adhesion parameter (A_Bi2WO6_/A_mica_) was calculated, yielding values in the range of ∼ 0.73–0.89 across the investigated frequency range. Despite small variations, this ratio remains within the same order of magnitude, indicating that adhesion decreases proportionally for both surfaces.

In addition, the separation between the main Gaussian peaks (ΔA) decreases from approximately ∼ 2.3 nN at 1 kHz to ∼ 1.2 nN at 4 kHz. These results indicate that the reduction in adhesion occurs in a correlated manner, preserving the relative contrast between the nanosheet and the substrate, while simultaneously decreasing the overall interaction strength. These results highlight the strong dependence of adhesion measurements on the oscillation frequency, which is relevant for reliable AFM characterization of nanoscale materials.

Since adhesion forces are composed of various physical interactions,^35^ we can analyze which of these contributions play a dominant role in the observed results. As the probes were not functionalized, contributions related to chemical bonding can be disregarded; these bonds refer to the ligand–receptor interactions in functionalized probes. Analyzing the contribution of capillary forces (or meniscus), we found that the probes used here have a nominal tip radius of 2 nm and a conical shape. The small contact angle formed at the tip–surface interface minimizes the meniscus effect. Additionally, Butt et al.^36^ point out that capillary forces are minimal under low-humidity conditions. By comparison of adhesion and topography maps, it is possible to observe that the strongest forces occur in different regions, not necessarily where holes are present. Therefore, we can consider that electrostatic and van der Waals forces significantly influence the adhesion results.

During the AFM scanning process, charge polarization of the tip may occur because of electrostatic interactions with charges accumulated on the sample surface. The aluminum atoms that compose the tip tend to acquire positive charges upon electrification, which influence the tip–sample interaction. The single-unit-cell Bi_2_WO_6_ layers exhibit a [BiO]^+^–[WO_4_]^2–^–[BiO]^+^ sandwich-like substructure. Within these layers, unit cells are arranged along the a and b directions, while the c parameter remains constant. As a result, the SUC-Bi_2_WO_6_ surface displays a distribution of positive charges that weaken the adhesion forces between the tip and the sample during scanning.

The freshly cleaved surface of mica is frequently used as a substrate for studying the aggregation behavior, interaction mechanisms, and bonding theories of biomolecules and semiconductor materials.^37^ Its lamellar crystalline structure includes a substitution of approximately one in four Si^4+^ ions by Al^3+^, giving rise to a net negative surface charge balanced by K^+^ ions within the layers. When mica is cleaved, the loss of these potassium ions leads to a highly negative surface potential, which strengthens electrostatic interactions and van der Waals forces with the AFM probe.^38,39^ In contrast, the surface of SUC-Bi_2_WO_6_, though polar due to the presence of Bi^3+^ and W^6+^ ions,^40^ has a crystalline structure that distributes charges more evenly, resulting in weaker electrostatic interactions. Additionally, Bi_2_WO_6_ exhibits lower electronic density and fewer active surface groups, which reduces the van der Waals forces compared to mica. These differences explain the stronger adhesion forces observed between the AFM tip and the mica.

AFM analysis confirmed the formation of a uniform SUC-Bi_2_WO_6_ layer and revealed nanoscale porelike surface defects. These observations highlight both the morphological sensitivity of the SUC-Bi_2_WO_6_ layer to measurement conditions and the intrinsic presence of structural discontinuities within the surface nanosheets. Such imperfections may compromise the electronic and structural continuity of the material, promoting charge carrier trapping and enhancing nonradiative recombination pathways, thereby limiting performance in optoelectronic and photocatalytic applications.^41,42^ However, similar surface features in two-dimensional Bi-based materials have been associated with the formation of additional active sites that enhance photocatalytic activity, particularly in reactions such as water oxidation and CO_2_ reduction.^43,44^ In Bi_2_WO_6_, a porous structure enriched with oxygen vacancies improves molecular adsorption, including that of tetracycline,^45^ while the controlled introduction of bismuth vacancies facilitates charge separation, further enhancing photocatalytic efficiency.^46^ Thus, although hole-type defects may present challenges depending on the application, they also offer opportunities for functional optimization in surface-sensitive technologies.^43^

#### 3.4.2 Variation of PeakForce

To evaluate the effect of varying the applied force on the SUC-Bi_2_WO_6_ nanosheets, the PeakForce was increased from 1 nN to 5 nN. Figure 7a–e demonstrates the topography maps of the SUC-Bi_2_WO_6_ nanosheet on mica. The side scale bar represents the height values of the scan and is given in nanometers. According to the color code of the topography maps, the mica substrate is represented by dark colors, while SUC-Bi_2_WO_6_ is represented by light colors (yellow). It is possible to observe that the nanosheet presents small holes on its surface. Using the statistical measurement tool of Gwyddion software, it was possible to measure the area and volume of these holes.

**Figure 7:**
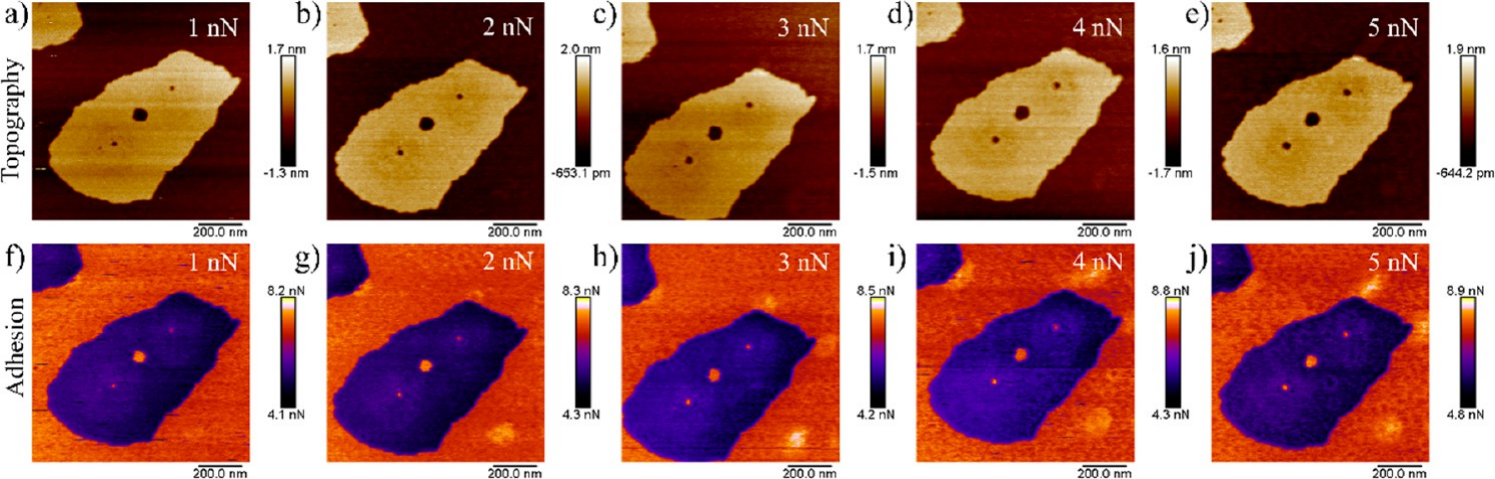
Topography maps (a–e) and adhesion forces (f–j) of the surface of the SUC-Bi_2_WO_6_ nanosheet on mica, varying the peak force from 1 nN to 5 nN, with a scanning area of 2.5 × 2.5 μm^2^. In the topographical maps, the light colors indicate the surface of the nanosheet, which has three holes that increase in area as the force applied by the probe increases. Light (dark) colors indicate higher (lower) adhesion strength in the adhesion maps.

The maps illustrated in Figure 7f–j represent the adhesive forces resulting from the tip–sample interaction. The side scale bar is given in forces on the order of nanonewtons. The color code of the images indicates regions of high adhesion with light colors (orange) and regions of low adhesion with dark colors (purple). As previously mentioned, the adhesion force F_ad_ is defined as the maximum force of attraction between the tip and the sample, a combination of different interactions.^35^ However, for our study, we can consider that mostly the interactions of electrostatic and van der Waals forces significantly influence the adhesion results.

Figure 8 illustrates the evolution of the pore area and volume on SUC-Bi_2_WO_6_ nanosheets as a function of PeakForce. As Figure 8a shows, the pore area increases linearly with the applied force, while the volume follows a nonlinear trend, well described by a third-degree polynomial. This indicates that although the lateral expansion is proportional to the applied force, the vertical deformation increases more rapidly. At 1 nN, the average area and volume of the holes were 4075 nm^2^ and 3411 nm^3^, respectively, increasing to 4470 nm^2^ and 5444 nm^3^ at 5 nN. Figure 8b supports this interpretation by showing representative height profiles at 1 nN and 5 nN, where the same holes exhibit greater depth and lateral expansion under higher force. The structural deformation observed at higher applied forces was irreversible, indicating a permanent nanosheet degradation. These results confirm that increased PeakForce does not create new holes but amplifies the deformation of existing ones, consistent with the behavior observed under the frequency variation.

**Figure 8:**
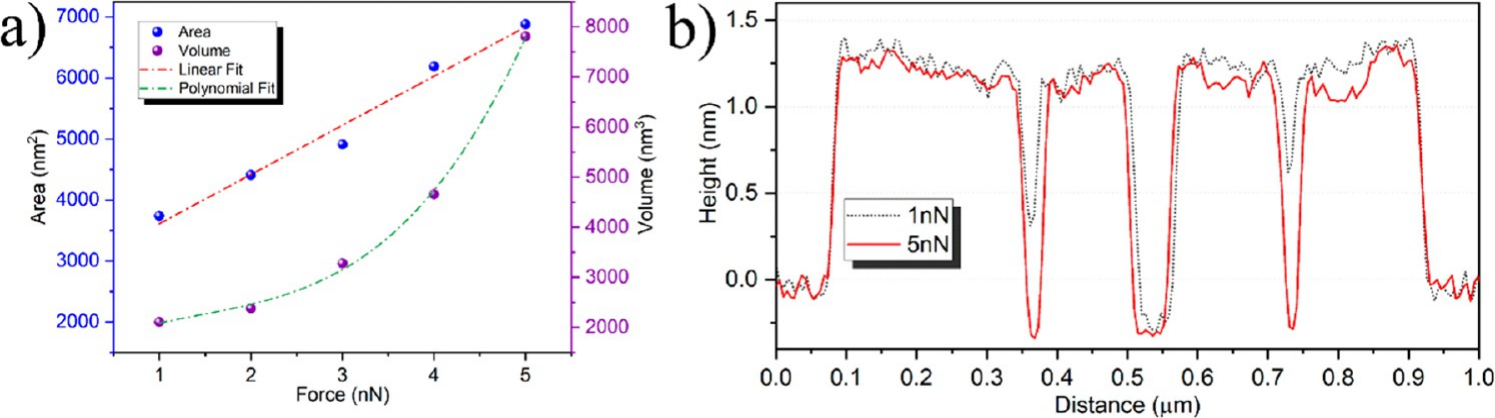
(a) Behavior of volume and area of holes on the surface of single-unit-cell layers Bi_2_WO_6_ as a function of indentation peak force. Area of holes increases linearly with force, reflecting proportional surface expansion. In contrast, volume grows polynomially due to a nonlinear increase in hole depth, combined with lateral expansion. (b) Cross-sectional profiles extracted from SUC-Bi_2_WO_6_ at 1 nN (black dashed line) and 5 nN (solid red line), showing deeper and wider holes at higher forces.

Figure 9a–e presents the statistical distribution of adhesion force values corresponding to the maps acquired under varying PeakForce, from 1 nN to 5 nN. Two well-defined adhesion populations are observed for all PeakForce values, corresponding to the mica substrate and the SUC-Bi_2_WO_6_ nanosheet. The mica substrate exhibits higher adhesion values, ranging from approximately 7.2 nN to 7.8 nN, while the SUC-Bi_2_WO_6_ nanosheet presents lower adhesion between approximately 5.0 nN and 6.1 nN. The average adhesion values and their respective statistical dispersions are detailed in Table 2.

**Figure 9:**
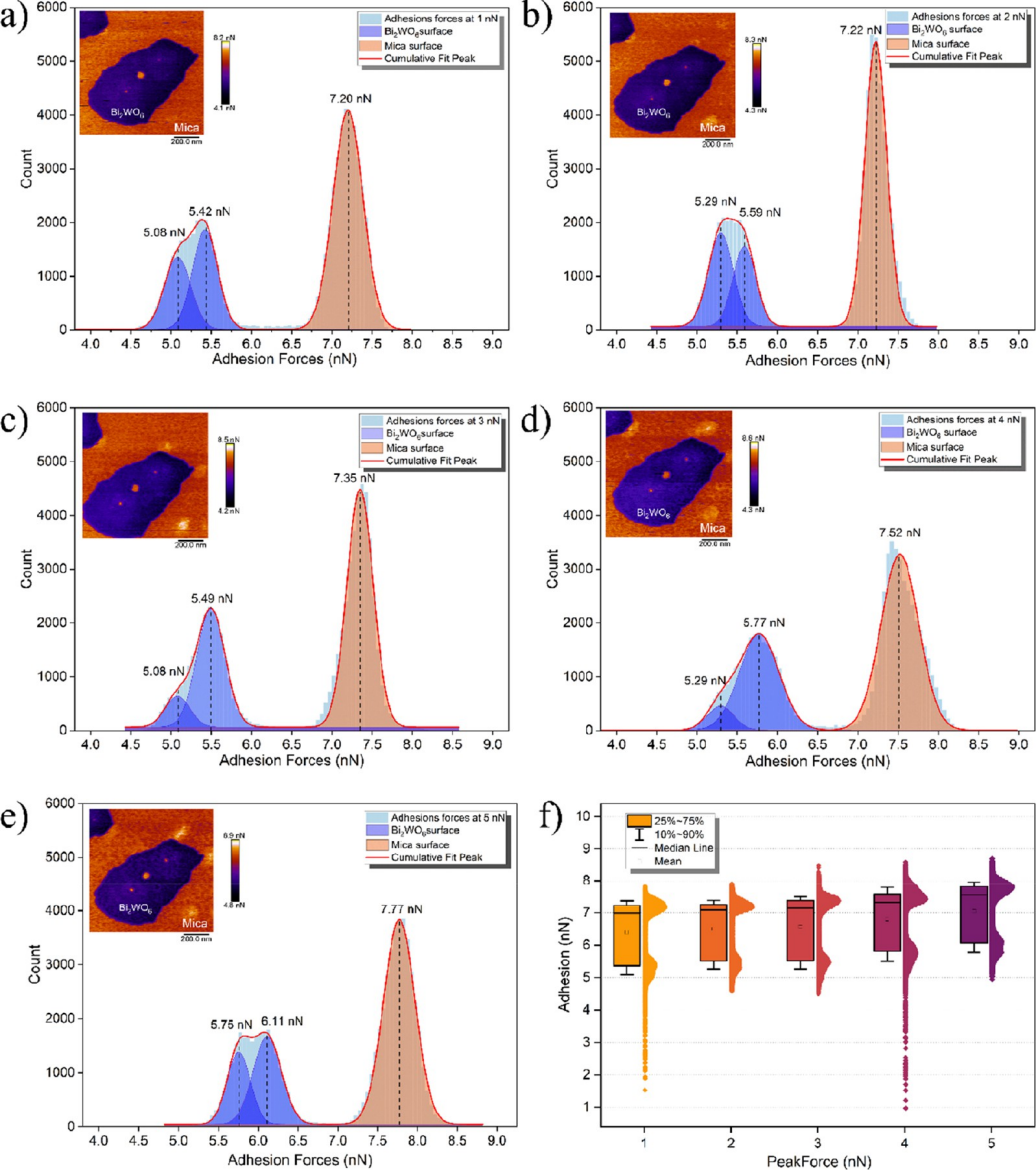
Statistical distribution of adhesion forces as a function of applied PeakForce (1 nN to 5 nN). (a–e) Histograms extracted from the adhesion maps (insets), exhibiting a bimodal distribution that separates the SUC-Bi_2_WO_6_ nanosheet (lower adhesion) from the mica substrate (higher adhesion). Solid lines represent the cumulative Gaussian fits, and vertical dashed lines indicate the mean adhesion for each population. (f) Violin and box plots summarizing the adhesion data showing a slight increase in adhesion values while preserving the contrast between the materials.

**Table 2:** Average Values of Adhesion Forces of the SUC-Bi_2_WO_6_ Nanosheet on Mica with PeakForce Variation

	PeakForce					
		1 nN	2 nN	3 nN	4 nN	5 nN
adhesion (nN)	Bi_2_WO_6_	5.08 ± 0.02	5.29 ± 0.06	5.08 ± 0.05	5.29 ± 0.01	5.57 ± 0.02
		5.42 ± 0.01	5.58 ± 0.06	5.49 ± 0.02	5.77 ± 0.01	6.11 ± 0.01
	Mica	7.19 ± 0.01	7.22 ± 0.01	7.35 ± 0.01	7.52 ± 0.01	7.77 ± 0.01

As PeakForce increases, a slight increase in adhesion is observed for both surfaces. This behavior is attributed to the increase in effective tip–sample contact area and the closer interfacial proximity achieved at higher scanning forces. Furthermore, since Bi_2_WO_6_ is a known piezoelectric material,^47^ the structural deformation induced by higher PeakForce may generate local surface charges. This effect could also contribute to the slight variations in adhesion forces. Despite this increase, the separation between the adhesion distributions remains approximately constant (∼1.75 nN), indicating that the adhesion contrast between mica and SUC-Bi_2_WO_6_ is highly stable with respect to variations in PeakForce.

The violin and box plot representation shown in Figure 9f confirms the statistical stability of adhesion values, with relatively small dispersion observed for both materials across the investigated force range. The consistency of the adhesion contrast suggests that the variation in the applied force primarily affects the absolute magnitude of the interaction without altering the intrinsic surface properties of the materials. Overall, the results demonstrate that the SUC-Bi_2_WO_6_ nanosheet maintains structural stability under increasing PeakForce while preserving a clear adhesion contrast relative to the mica substrate. These findings confirm that PeakForce QNM measurements provide reliable and reproducible morphological and adhesive characterization of the nanosheet-substrate system.

### 3.5 Characterization via KPFM

To further investigate the surface potential of the SUC-Bi_2_WO_6_ nanosheets, Kelvin probe force microscopy (KPFM) was employed to simultaneously map topography, adhesion, and surface potential, helping to understand how the nanosheet geometry affects the charge distribution.

Figure 10 provides three-dimensional maps of topography (a), adhesion (d), and surface potential (g) for the same region of the SUC-Bi_2_WO_6_ nanosheets deposited on mica, obtained simultaneously by KPFM. The dashed white square highlights a magnified area containing a single-unit-cell Bi_2_WO_6_ layer with an elongated morphology of approximately 800 nm, shown in images (b), (e), and (h). Cross-sectional profiles extracted along the dashed lines appear in images (c), (f), and (i), providing detailed comparisons across the nanosheet surface.

**Figure 10:**
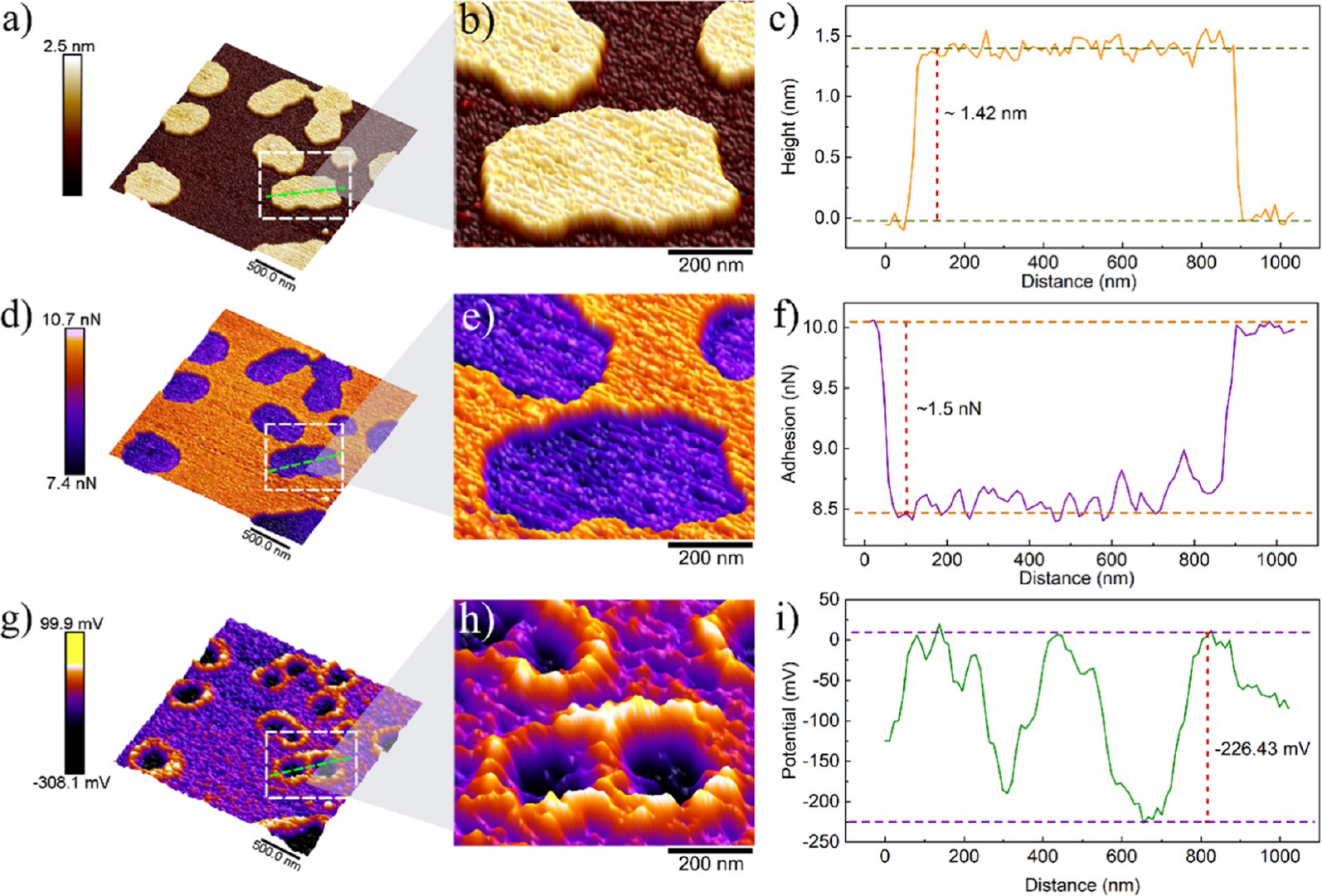
Three-dimensional maps of topography (a), adhesion forces (d), and surface potential (g) of a SUC-Bi_2_WO_6_ nanosheet on mica. The dashed white square delimits the magnification region shown in the images (b, e, h), highlighting an elongated structure (∼800 nm in length). The corresponding cross-sectional profiles (c, f, i) describe the uniform topography, adhesion contrast, and a surface potential distribution revealing a high potential central region that divides the nanosheets into two domains.

As expected, the topographic profiles confirm a height of approximately 1.42 nm (Figure 10a–c). The adhesion map (Figure 10d–f) reveals a clear contrast between the SUC-Bi_2_WO_6_ surface and the mica substrate, with the latter exhibiting a higher adhesion force with the probe, showing an average difference of ∼ 1.5 nN compared to the nanosheet surface. The surface potential map (Figure 10g–i) reveals that the SUC-Bi_2_WO_6_ nanosheet edges consistently exhibit higher V_CPD_ than the central region, forming a surface potential well with a ΔV_CPD_ of −226.43 mV (Figure 10h). In addition to intrinsic electronic effects, the higher V_CPD_ at the edges may also be partially influenced by the interaction between the SUC-Bi_2_WO_6_ nanosheet and the mica substrate, whose surface dipoles modify the local electrostatic environment.^48^ Interestingly, a high potential is also observed at the center of the elongated SUC-Bi_2_WO_6_ nanosheets, dividing the structure into two nearly circular domains, similar to individual circular nanosheets. The polycrystalline nature of these nanosheets revealed by TEM suggests that this central high potential region may be associated with a grain boundary. The localized increase in surface potential is consistent with the surface potential behavior of grain boundaries in polycrystalline semiconductor materials.^49^

A second scan from a different region of the sample (Figure 11) shows a similar topography (a) and adhesion force (d) but again with a geometry-dependent surface potential distribution (g). In this region, a circular nanosheet of approximately 600 nm in diameter is observed (Figure 11b,e,h). As in the previous case, the topographic (Figure 11c) and adhesion (Figure 11f) profiles do not depend on the nanosheet shape, but the same cannot be said for the surface potential distribution.

**Figure 11:**
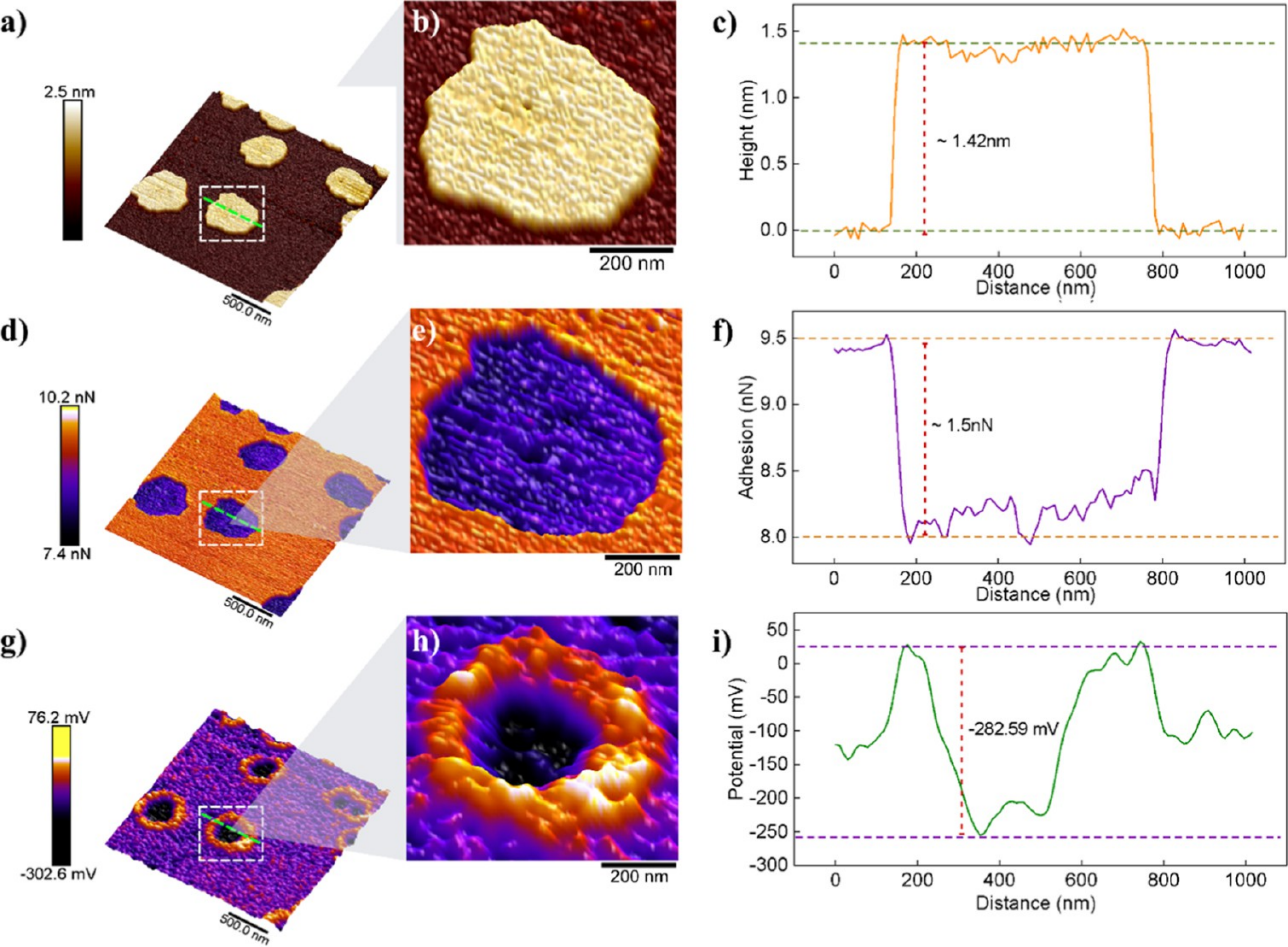
KPFM analysis of a different sample region featuring a circular SUC-Bi_2_WO_6_ nanosheet (∼600 nm in diameter). Three-dimensional maps illustrate the topography (a), adhesion forces (d), and surface potential (g). The dashed white square delimits the magnification areas detailed in (b, e, h). The corresponding cross-sectional profiles (c, f, i) highlight the uniform height, adhesive contrast, and the characteristic higher surface potential at edges, forming a distinct potential well in this circular geometry.

The surface potential map of the circular nanosheet displays higher V_CPD_ values at the edges and lower values at the center, resulting in a potential difference of approximately −282.59 mV. To quantitatively support these observations, a statistical histogram analysis of the surface potential was performed for both morphologies. Figure 12 presents the potential histograms extracted from the KPFM maps of the circular and elongated nanosheets, fitted with multiple Gaussian components. The corresponding work function (Φ) values, calculated using a calibrated tip (Φ_tip_ = 4.601 eV, see the Supporting Information for calibration details), are summarized in Table 3.

**Figure 12:**
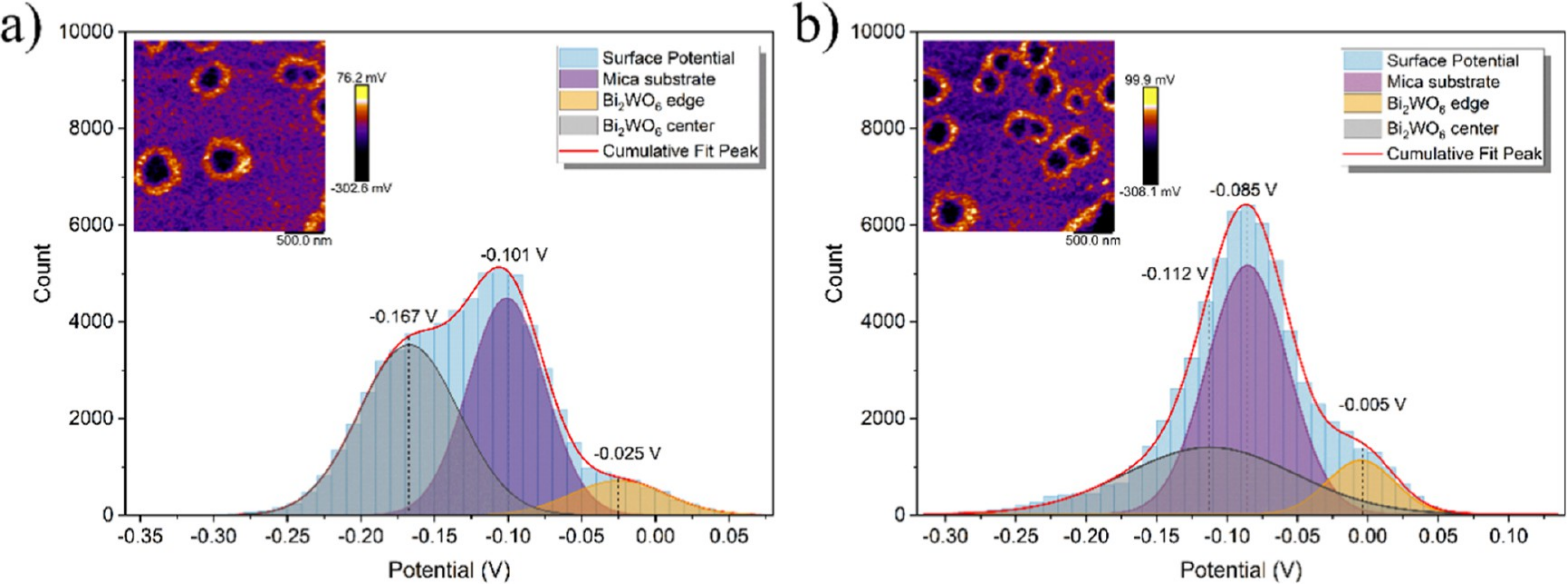
Surface potential histograms of SUC-Bi_2_WO_6_ nanosheets on mica for (a) circular and (b) elongated morphologies. Histograms extracted from the respective KPFM fitted with multiple Gaussian components, where the peaks correspond to distinct surface potential regions. The gray components are associated with the nanosheet center, the orange components correspond to the nanosheet edges, and the purple components correspond to the mica substrate. The peak center values (V_CPD_) are indicated for each region.

**Table 3:** Surface Potential and Calculated Work Function (Φ) for the Circular and Elongated SUC-Bi_2_WO_6_ Nanosheets The Work Function Was Calculated Using the Sample Bias Formula, with a Calibrated Tip Work Function (Φ_tip_ = 4.601 eV)

morphology	region	V_CPD_ (peak Center, V)	work function (Φ, eV)
circular	nanosheet edges	–0.022 ± 0.003	4.579 ± 0.003
	mica substrate	–0.101 ± 0.001	4.500 ± 0.001
	nanosheet center	–0.168 ± 0.001	4.433 ± 0.001
elongated	nanosheet edges	–0.005 ± 0.004	4.596 ± 0.004
	mica substrate	–0.085 ± 0.001	4.516 ± 0.001
	nanosheet center	–0.112 ± 0.037	4.489 ± 0.037

For the circular geometry (Figure 12a), the histogram confirms a well-defined surface-potential distribution. The central domain presents the lowest work function (4.433 eV), establishing a distinct potential well relative to that of the edges (4.579 eV). This potential distribution suggests that the circular nanosheet represents a highly stable, low-energy configuration with a well-defined charge distribution. The higher work function at the edges is likely driven by broken bonds, which alters the local electrostatic environment. In contrast, the histogram for the elongated nanosheets (Figure 12b) reveals a broader potential distribution for the central region along with a slightly higher average work function (4.489 eV). This statistical broadening is possibly a consequence of the inhomogeneous charge distribution introduced by structural features such as grain boundaries. These experimental observations align with first-principles calculations for 2D Bi_2_WO_6_, which predict that reduced dimensionality induces spatial charge separation and high carrier mobility from the central regions toward the structural boundaries.^50^

The KPFM results provide important insights into the potential functional performance of SUC-Bi2WO6 nanosheets. The pronounced surface-potential heterogeneity observed between the nanosheet center and edges, reaching potential differences of approximately 226–283 mV, indicates the presence of intrinsic local electric fields capable of promoting charge separation and directional carrier migration. Such behavior is highly desirable for photocatalytic and photoelectrochemical applications, where efficient suppression of electron–hole recombination is essential.^51–55^ In addition, the higher work-function regions located at the nanosheet edges suggest preferential sites for charge accumulation and interfacial reactions. The ultrathin single-unit-cell thickness (∼1.42 nm) further minimizes carrier transport distances, while the nanoscale surface holes identified by AFM may increase the density of active sites and enhance adsorption processes. Collectively, these characteristics suggest that SUC-Bi2WO6 nanosheets possess a favorable nanoscale architecture for applications involving photocatalysis, photoelectrochemistry, sensing, and charge-transfer processes.^15^

It is worth noting that KPFM measurements are sensitive to environmental conditions and instrumental parameters, and if performed in controlled atmospheres or liquid environments, they could provide deeper insights into electrochemical activity and susceptibility to chemical reactions.^56^ These findings offer valuable insights into the structural, adhesive, and electrostatic properties of bismuth tungstate at the nanoscale, contributing to a more comprehensive characterization of its behavior and potential applications in electronic devices. Future studies should further explore the influence of synthesis conditions on nanosheet stability and investigate strategies for morphological control and the enhancement of their functional properties.

## 4 Conclusion

In summary, this study presents a comprehensive characterization of single-unit-cell-layer bismuth tungstate (SUC-Bi_2_WO_6_) nanosheets synthesized via hydrothermal methods. Structural analyses using powder X-ray diffraction (PXRD), Raman spectroscopy, and TEM confirmed the successful synthesis, revealing a polycrystalline lamellar morphology, an orthorhombic phase, and features indicative of reduced crystallite size. AFM revealed a uniform nanosheet thickness of approximately 1.40 nm, consistent with the expected value for a single-unit-cell layer. Morphological and adhesive analyses revealed that increasing the oscillation frequency promotes the emergence and expansion of nanoscale holes, suggesting localized structural degradation. The gradual increase of PeakForce further enlarged the holes, although the overall structural integrity remained stable at higher forces. KPFM measurements revealed that the morphology of the nanosheets strongly influenced their surface potential distribution. Circular nanosheets showed a more stable and well-defined potential profile, characterized by a lower central work function, suggesting a lower-energy configuration. In contrast, elongated structures exhibited a central high-potential region, corresponding to a grain boundary. This localized structural defect disrupts the uniform potential, indicating that they may fragment into more stable circular domains over time. Overall, this work provides valuable insights into the structure–property relationships of SUC-Bi_2_WO_6_ at the nanoscale. The material shows promise for applications that are sensitive to surface conditions and local work function variations such as photocatalysis and surface-mediated reactions. Additionally, this material may become more structurally robust and functionally tunable when combined with other 2D systems or polymeric matrixes, expanding its applications in photocatalysis and nanoelectronics.

## Supplementary Material



## Notes

KPFM probe calibration details, including AFM/KPFM characterization of the Al–Si–Au reference sample, Gaussian fitting parameters, determination of the probe work function, and validation of the calibration procedure (PDF)
