# Supplementary material for: Hydrothermal Synthesis and Nanoscale Characterization of Single-Unit-Cell Bi2WO6 Nanosheets: Correlation between Morphology, Adhesion, and Surface Potential
Source: ACS Omega. 2026 Jul 7;11(28):42909–22. doi: 10.1021/acsomega.6c05132 (PMC13393362; doi:10.1021/acsomega.6c05132)
Supplement: Supplementary file 1 [file ao6c05132_si_001.pdf]

Supplementary Information

Hydrothermal synthesis and nanoscale characterization of single-unit-cell Bi_2WO_6 nanosheets: Correlation between morphology, adhesion and surface potential

Charles Duarte Almeida [a*], Beatriz da Silva Batista [b], Madson Emanuel Vieira Mendonça [a], Dyego Maia de Oliveira [c], Eduardo Padrón-Hernández [c], Clenilton Costa dos Santos [a], Alan Silva de Menezes [a], Luciana Magalhães Rebelo Alencar [b], João Victor Barbosa Moura [a**]

[a] *Programa de Pós-graduação em Física, Universidade Federal do Maranhão, São Luís, 65080-805, MA, Brasil.*
[b] *Laboratório de Biofísica e Nanosistemas, Universidade Federal do Maranhão, São Luís, 65080-805, MA, Brasil.*
[c] *Departamento de Física, Universidade Federal de Pernambuco, Recife, 50740-540, PE, Brasil.*

S1 KPFM Tip Calibration

This section provides the quantitative calibration of the KPFM probe work function Φ_{tip}. An Al-Si-Au standard sample was used to establish the relationship between the measured contact potential difference V_{CPD} and the surface work function under the sample bias configuration.

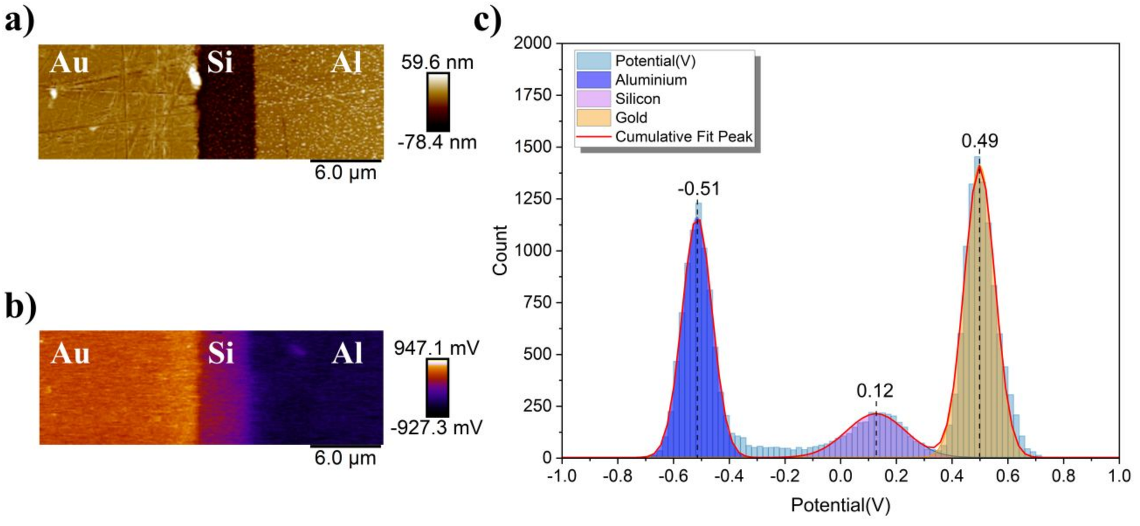

Figure S1: a) AFM topography and (b) KPFM potential map of the Al-Si-Au calibration standard. (c) Histogram of the potential distribution fitted with three Gaussian components.

To calibrate the KPFM probe work function, a standard Al-Si-Au sample was scanned. The potential distribution was fitted with three distinct Gaussian components, corresponding to each material on the grid, as detailed in Table S1. The high correlation coefficient ($R^2 > 0.99$) confirms the reliability of the fitting. The calibration was validated by calculating the work functions of Al and Si, which showed deviations of less than 0.02 eV from theoretical values.

Table S1: Gaussian fitting parameters extracted from the calibration standard histogram shown in Figure S1. Adjusted R-square = 0.99049. The tip work function (Φ_{tip}) was determined as 4.601 eV based on the Au reference (Φ_{Au} = 5.10 eV).

Peak	Material	Xc (Center, V)	w (Width, V)	A (Area)
**Peak 1**	Aluminium (Al)	-0.5152 ± 0.0006	0.1266 ± 0.0015	156.8 ± 1.59
**Peak 2**	Silicon (Si)	0.1269 ± 0.0048	0.2495 ± 0.0116	56.2 ± 2.29
**Peak 3**	Gold (Au)	0.4986 ± 0.0005	0.1299 ± 0.0012	194.6 ± 1.63

S2 Determination of the Tip Work Function Φ_{tip}

The quantitative calibration of the KPFM probe was performed using a standard Al-Si-Au sample. Under the Sample Bias configurations, the relationship between the measured contact potential difference (V_{CPD}) and the work functions is given by:

$$\Phi_{sample} = \Phi_{tip} + e \cdot V_{CPD} \quad \text{(S1)}$$

Rearranging the equation to solve for the tip work function (Φ_{tip}):

$$\Phi_{tip} = \Phi_{sample} - e \cdot V_{CPD} \quad \text{(S2)}$$

Using the Gold (Au) peak as the primary reference (Φ_{Au} = 5.10 eV) and the experimental value obtained from the Gaussian fit, the calculation is as follows:

$$\Phi_{tip} = 5.10\ eV - 0.4986\ eV = 4.601\ eV \quad \text{(S3)}$$

To ensure the accuracy of the determined Φ_{tip} the work function of the secondary standards (Al and Si) present in the same calibration grid were calculate and compared with literature values:

- Aluminium (Al):

$$\Phi_{Al} = \Phi_{tip} + e \cdot V_{Al} = 4.601\ eV + (-0.5152\ eV) = 4.086\ eV \quad \text{(S4)}$$

- Silicon (Si):

$$\Phi_{Si} = \Phi_{tip} + e \cdot V_{Si} = 4.601\ eV + 0.1269\ eV = 4.728\ eV \quad \text{(S5)}$$

The calculated work functions for Al (4.086 eV) and Si (4.728 eV) are in excellent agreement with standard literature values, which typically report ~4.08 eV for aluminum and 4.60–4.85 eV for doped silicon. This minimal deviation (< 0.02 eV for Al) provides robust validation of the determined tip work function (Φ_{tip}= 4.601 eV) and confirms the correct assignment of the Sample Bias polarity. Consequently, this rigorous quantitative calibration ensures the high reliability of the surface potential measurements and the subsequent work function extraction performed on the SUC-Bi_2WO_6 nanosheets discussed in the main manuscript.
